# Diphtheria: A novel cause of concern for Pakistan

**DOI:** 10.7189/jogh.13.03038

**Published:** 2023-09-01

**Authors:** Muhammad Hamza Shuja, Firzah Shakil, Hamza Imran, Mubashira Feroz

**Affiliations:** Department of Medicine, Dow University of Health Sciences, Baba-e-Urdu Road, Saddar, Karachi, Pakistan

Diphtheria is a serious bacterial infection most often caused by *Corynebacterium diphtheriae* [1]. It spreads into the mucous membranes of the throat, producing a diphtheria toxin, a bacterial exotoxin that kills cells by preventing protein synthesis [2]. The toxin impairs the lining of the throat and creates a false membrane, a typical feature of throat-, nose-, and tonsils-related diphtheria [2]. Occasionally, toxic strains of *C. ulcerans* and *C. pseudotuberculosis* also cause diphtheria [1]. Typically, bacterial culture followed by tests for detecting enzymes and toxins validates the clinical diagnosis after isolating and identifying the accountable *Corynebacterium diphtheriae* [3].

## SIGNS AND SYMPTOMS OF DIPHTHERIA

**Figure 1:**
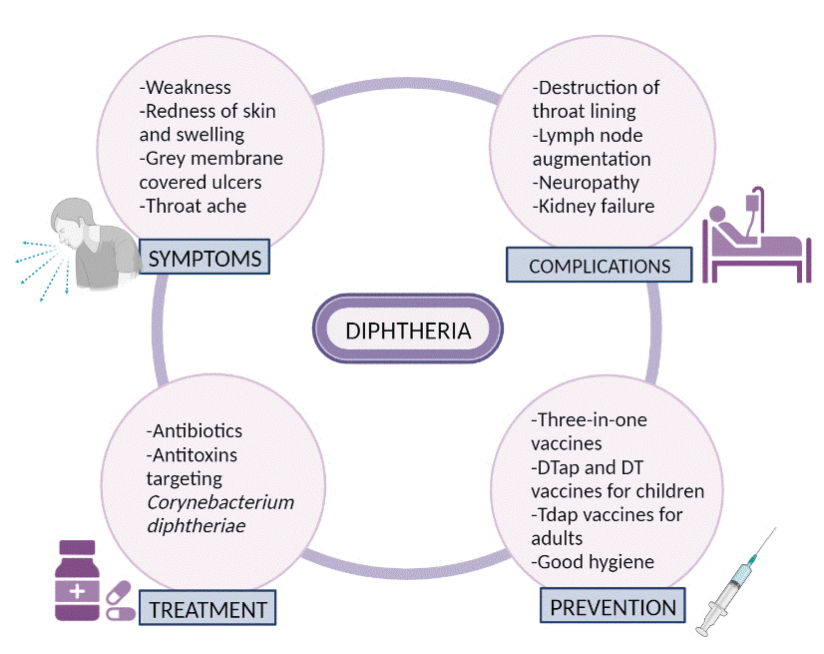
Summary of diphtheria.

The disease's distinctive signs and symptoms normally show up two to five days after infection [2], the most typical being hoarseness, a sore throat, and a thick, grey membrane covering the tonsils and throat. Additionally, rapid or difficult breathing and enlarged lymph nodes in the neck may develop [1]. In severe cases, after circulating in the blood or lymphatic system, the diphtheria toxin can invade outlying organs, potentially leading to nerve damage, renal failure, and inflammation of the heart myocardium in its later stages, especially in young children, where it can be fatal even with treatment [3]. However, some individuals who contract diphtheria-causing germs only develop a mild illness or none at all [2]. In most cases, diphtheria affects the nose and throat mucous membranes. It can also impact the skin, producing discomfort, redness, and swelling in cases of bacterial skin infections, and gray membrane-covered ulcers [2]. People who have poor hygiene and live in cramped quarters are typically more susceptible to the disease, which is usually transmitted through airborne means, and more rarely via touch, an organism’s liquids, or contact with an infected wound (**Figure 1**) [2].

## PREVALENCE AND OUTBREAKS IN DEVELOPING COUNTRIES

Diphtheria is relatively rare in the USA and other developed countries due to immunisation programs and is more prevalent in those with inadequate medical services or immunisation alternatives (such as homoeopathic remedies) [2]. Despite the availability of effective vaccines, diphtheria could reappear in nations with unmaintained recommended immunisation regimens and rising numbers of adults at risk of infection; for example, many countries in Asia and Africa still have many outbreaks and cases annually [1]. Recent outbreaks linked to displaced populations and infrastructure issues highlight the ongoing dangers of diphtheria, such as the November 2017 outbreak among Rohingya refugees in the Kutupalong camp in Bangladesh, where 8740 cases and 45 fatalities have been recorded by June 2019 [4]. The Bangladesh outbreak was preceded by others in Venezuela (1904 suspected cases, 164 fatalities), Yemen (1907 suspected cases, 98 fatalities), and Haiti (808 probable cases, 107 fatalities) [4]. In the 1990s, a decade-long outbreak in Eastern Europe that resulted in 157 000 cases and 5000 fatalities grew into a global epidemic [4].

## VACCINATION CHALLENGES IN DEVELOPING COUNTRIES

Diphtheria was a common disease in young children before the development of antibiotics [5]. Today, a vaccine makes the illness not only treatable, but also preventable. Immunisation against tetanus, pertussis, and diphtheria is frequently done through one vaccination; its most recent iteration, referred to as the diphtheria, tetanus, and pertussis (DTaP) vaccine, is often advised by doctors in the USA for infants [5]. Children under the age of seven can get vaccinated against the disease with these vaccines, which are commonly available as Daptacel, Quadracel, and Vaxelis. They can also be given diphtheria (DT) vaccines, which only fight against diphtheria and tetanus [6]. Another iteration is the tetanus, diphtheria, and pertussis (Tdap) vaccine (Adacel, Boostrix) for adults and older adolescents [5], which is also advised during pregnancy [6]. However, adults, like children, can also be given tetanus and diphtheria (Td) vaccines, as they do not get whooping cough as frequently as infants do [6]. The vaccination of children in Pakistan, however, has always been a challenge, as approximately three million children annually are denied access to basic immunisation [7]. The proportion of vaccination coverage varies significantly among various provinces [7]. Common causes of partial or non-immunisation of children include a lack of knowledge, fear of potential side effects, a lack of vaccines or vaccine providers, and a lack of communication between health care professionals and primary caregivers [8]. This causes a subsequent knowledge gap about follow-up vaccines and their significance.

## RECENT OUTBREAK IN PAKISTAN AND THE WAY FORWARD

Since 2016, there has been a steady rise in the number of cases of diphtheria recorded worldwide, which doubled from an average of 8105 cases between 1997 and 2017 to 16 651 in 2018 [9]. Based on recent reports, the National Institutes of Health (NIH) has issued an alert regarding a potential diphtheria outbreak in Pakistan [10] due to the rising number of cases in the winter months from November 2022 to February 2023 [10]. As of February 2023, about 26 cases of diphtheria were reported, with the warning of a need to suppress the spread of the bacterial infection [10]. However, as highlighted by Siddiqi et al. [11], there is low coverage, awareness, and knowledge of the third dose of the diphtheria-pertussis-tetanus vaccine (DPT3) in Pakistan. Moreover, On 27 September 2022, the southern Pakistani state of Sindh recorded 10 diphtheria deaths in the last two months; the true death toll may be five times greater, yet even these official numbers are a cause for concern [12]. Consequently, health authorities are in fear and anticipation of a more widespread and deadly wave of diphtheria if sufficient precautionary measures are not taken [12].

**Figure:**
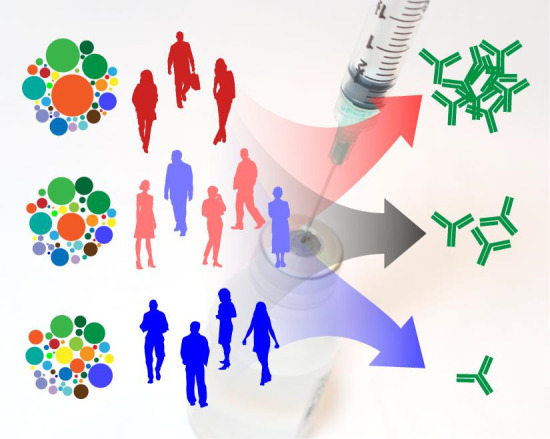
Photo: Vaccination against the bacteria can be pivotal in preventing diphtheria. Source: National Institutes of Health, National Institute of Allergy and Infectious Diseases. Available: https://www.flickr.com/photos/54591706@N02/14165434229. Licensed under CC BY 2.0 license.

To contain outbreaks, Pakistan requires more diphtheria-pertussis-tetanus (DPT) booster vaccines in its Expanded program on immunization (EPI) program along with higher rates of routine childhood immunisation, a task made difficult by the large immunisation coverage area [12]. Diphtheria cases are common in Pakistan, but because general practitioners lack the necessary training and experience, they are only occasionally referred to specialised hospitals before they have reached an irreversible stage, where even an antitoxin proves ineffective [12]. Pakistan must develop a comprehensive immunisation program covering every area in every province if it wants to entirely eradicate this illness and stop any subsequent outbreaks. To prevent diphtheria from becoming fatal, doctors should be trained to recognise early warning signs and take timely action, while public service announcements emphasising hygiene and proper waste disposal should be made, and sanitary goods should be distributed to rural and flood-affected communities.

## References

[1] Sharma NC Efstratiou A Mokrousov I Mutreja A Das B Ramamurthy T Diphtheria. Nat Rev Dis Primers. 2019;5:81. 10.1038/s41572-019-0131-y 31804499

[2] Kim DK Hunter P Advisory Committee on Immunization Practices Recommended Adult Immunization Schedule, United States, 2019. Ann Intern Med. 2019;170:182-92. 10.7326/M18-3600 30716757

[3] Doerr S. Diphtheria. 2022. Available: https://www.emedicinehealth.com/diphtheria/article_em.htm. Accessed: 3 March 2023.

[4] Truelove SA Keegan LT Moss WJ Chaisson LH Macher E Azman AS Clinical and Epidemiological Aspects of Diphtheria: A Systematic Review and Pooled Analysis. Clin Infect Dis. 2020;71:89-97. 10.1093/cid/ciz808 31425581 PMC7312233

[5] Centers for Disease Control and Prevention. Diphtheria. Diagnosis, Treatment, and Complications. 2022. Available: https://www.cdc.gov/diphtheria/about/diagnosis-treatment.html. Accessed: 3 March 2023.

[6] Cleveland Clinic. Diphtheria Vaccine. 2021. Available: https://my.clevelandclinic.org/health/drugs/21565-diphtheria-vaccine. Accessed: 3 March 2023.

[7] World Health Organization Regional Office for the Eastern Mediterranean. Immunization leaders call for increased political support for immunization in Pakistan. 2023. Available: https://www.emro.who.int/media/news/political-support-immunization-pakistan.html. Accessed: 3 March 2023.

[8] Riaz A Husain S Yousafzai MT Nisar I Shaheen F Mahesar W Reasons for non-vaccination and incomplete vaccinations among children in Pakistan. Vaccine. 2018;36:5288-93. 10.1016/j.vaccine.2018.07.024 30054162

[9] Will RC Ramamurthy T Sharma NC Veeraraghavan B Sangal L Haldar P Spatiotemporal persistence of multiple, diverse clades and toxins of *Corynebacterium diphtheriae.* Nat Commun. 2021;12:1500. 10.1038/s41467-021-21870-5 33686077 PMC7940655

[10] Hassan T. NIH warns of Diphtheria outbreak in Pakistan. 2023. Available: https://arynews.tv/nih-warns-of-diphtheria-outbreak-in-pakistan/. Accessed: 3 March 2023.

[11] Siddiqui AA Khan M Khan JA Haseeb SS Mohib A Kadri HM Awareness, Knowledge, and Coverage of Vaccination Against Tetanus, Diphtheria, and Pertussis Among Medical Students of Karachi: A Cross-sectional Analysis. Cureus. 2019;11:e4472. 10.7759/cureus.4472 31249750 PMC6579328

[12] Arab News. After floods, diphtheria deaths raise alarm in southern Pakistan. 2022. Available: https://arab.news/nyghz. Accessed: 3 March 2023.

